# A genome assembly of the Atlantic chub mackerel (*Scomber colias*): a valuable teleost fishing resource

**DOI:** 10.46471/gigabyte.40

**Published:** 2022-02-14

**Authors:** André M. Machado, André Gomes-dos-Santos, Miguel M. Fonseca, Rute R. da Fonseca, Ana Veríssimo, Mónica Felício, Ricardo Capela, Nélson Alves, Miguel Santos, Filipe Salvador-Caramelo, Marcos Domingues, Raquel Ruivo, Elsa Froufe, L. Filipe C. Castro

**Affiliations:** ^1^ CIIMAR – Interdisciplinary Centre of Marine and Environmental Research, U. Porto – University of Porto, Porto, Portugal; ^2^ Department of Biology, Faculty of Sciences, U. Porto - University of Porto, Portugal; ^3^ Center for Global Mountain Biodiversity, GLOBE Institute, University of Copenhagen, Copenhagen, Denmark; ^4^ Center for Macroecology, Evolution, and Climate, GLOBE Institute, University of Copenhagen, Denmark; ^5^ CIBIO - Centro de Investigação em Biodiversidade e Recursos Genéticos, InBIO - Laboratório Associado, Campus de Vairão, Universidade do Porto, 4485-661 Vairão, Portugal; ^6^ BIOPOLIS - Program in Genomics, Biodiversity and Land Planning, CIBIO, Campus de Vairão, 4485-661 Vairão, Portugal; ^7^ Portuguese Institute for the Sea and Atmosphere, I.P. (IPMA), Portugal

## Abstract

The Atlantic chub mackerel, *Scomber colias* (Gmelin, 1789), is a medium-sized pelagic fish with substantial importance in the fisheries of the Atlantic Ocean and the Mediterranean Sea. Over the past decade, this species has gained special relevance, being one of the main targets of pelagic fisheries in the NE Atlantic. Here, we sequenced and annotated the first high-quality draft genome assembly of *S. colias*, produced with PacBio HiFi long reads and Illumina paired-end short reads. The estimated genome size is 814 Mbp, distributed into 2,028 scaffolds and 2,093 contigs with an N50 length of 4.19 and 3.34 Mbp, respectively. We annotated 27,675 protein-coding genes and the BUSCO analyses indicated high completeness, with 97.3% of the single-copy orthologs in the Actinopterygii library profile. The present genome assembly represents a valuable resource to address the biology and management of this relevant fishery. Finally, this genome assembly ranks fourth in high-quality genome assemblies within the order Scombriformes and first in the genus *Scomber*.

## Data description

### Background and context

**Figure 1.**
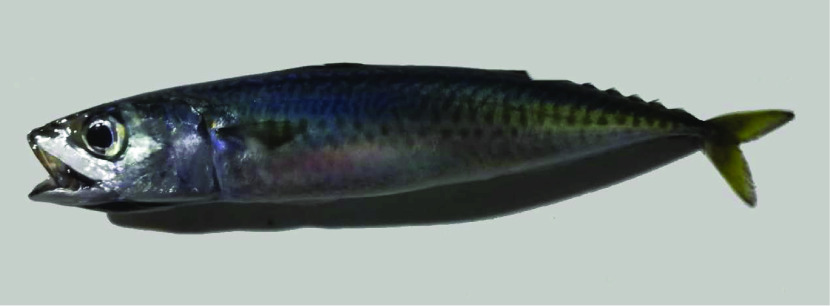
Photograph of Atlantic chub mackerel, *Scomber colias*. The specimen was caught in 2020 and used for Pacbio HiFi genome assembly.

The family Scombridae is divided into 2 subfamilies (Gasterochismatinae and Scombrinae), with 15 genera and around 49 described species, comprising mackerels, bonitos, and tunas [1]. The representative genus of the Scombridae, i.e., *Scomber*, includes 4 species: *S. scombrus*, *S. japonicus*, *S. australasicus*, and *S. colias*. The Atlantic chub mackerel, *Scomber colias* (Gmelin, 1789) (NCBI:txid338315, FishBase ID:54736), is a small coastal pelagic fish that is distributed widely, being found in the Atlantic Ocean from the Bay of Biscay to South Africa (including the Canary, Madeira, Azores, and Saint Helena Islands), and in the Mediterranean Sea (Figure 1) [2]. *Scomber colias* is usually found at depths of up to 300 m and occupies a key position in the trophic web. This species acts as a link between primary producers and top predators, since it feeds mainly on zooplankton and small pelagic fish, and is an essential element of the diet of larger pelagic fish (e.g., tuna, swordfish, and sharks) and marine mammals (e.g., dolphins and seals) [3]. Besides its ecological importance, *S. colias* also supports important commercial fisheries for several countries across its distribution range, being an important component in the diet of local populations [1, 4]. This is probably related to its nutritional value, as this mackerel is a valued source of important fatty acids for human nutrition, particularly docosahexaenoic acid (DHA), an omega-3 fatty acid [5, 6]. Additionally, *S. colias* is used as bait for the tuna longline and handline fisheries, and is caught in purse seine and pelagic trawl fisheries which target sardines and anchovies [7].

 The availability of *S. colias* makes it a sustainable marine resource [6] and a viable alternative to the European sardine (*Sardina pilchardus*), which is under fishing restrictions due to a population decline. Curiously, fluctuations in abundance and a northwards shift in the distribution of *S. colias*, with a likely inverse relationship with sardine abundance, have ﻿been recently demonstrated [8]. Due to its ecological and economic importance, *S. colias* has been the focus of several recent studies on different aspects of its fisheries and biology [3, 8, 9]. Yet, genomic resources for the species are still limited. Presently a (liver) transcriptome [10], a mitogenome  [11], and single-nucleotide polymorphism (SNP) data obtained through restriction site-associated DNA sequencing [12], have been described for the species. With the vast majority of the world’s fish stocks already in collapse, and with climate change as additional pressure, information on fish genomes is becoming a pressing tool to address conservation efforts [13, 14]. Here, we report the first high-quality draft genome of *S. colias*, assembled with Illumina and Pacific Biosciences (PacBio) Single Molecule High-Fidelity (HiFi) reads. This resource provides a critical platform to uncover the species’ adaptive physiological potential in a changing environment. Specifically, it will help understand the current observed populational northward shift, postulated to be part of a more general expansion of species from warmer areas [8]. Moreover, being one of the genomes with higher quality within the family Scombridae and the first within the *Scomber* genus, this information will help to improve the conservation, management, and sustainable exploitation of this valuable fish resource as well as that of its highly valued congeners.

## Methods

### Sampling and DNA extraction

Two specimens of *S. colias* were collected at 2 sampling time points. The first specimen was collected in 2017, during the “*Programa Nacional de Amostragem Biológica*” managed by the Instituto Português do Mar e da Atmosfera” (IPMA), in North Atlantic waters (41.501944 N 8.851667 W). From this individual, 2 tissue types were collected, and were stored in 100% ethanol (muscle) or RNA *later* (liver). Liver tissue was used to produce and describe the first liver transcriptome of *S. colias* [10]. Muscle tissue was used in the present study, for genomic DNA (gDNA) extraction using the DNeasy Blood and Tissue Kit (Qiagen, Hilden, Germany), following the manufacturer’s instructions. The gDNA was then used for Illumina paired-end (PE) sequencing (described below). The second specimen was caught in 2020, near Mira, Portugal (40.5588270 N 9.4529720 W). Immediately upon harvesting, the muscle was snap frozen in liquid nitrogen. The frozen tissue was shipped to Brigham Young University DNA Sequencing Center (BYU), where gDNA with high molecular weight was extracted from 1.1 g of muscle using the QIAGEN Genomic-tip 20/G kit. The quality and concentration of the gDNA were assessed with Qubit Fluorometric system (ThermoFisher), and the fragment size was determined with a fragment analyser (Agilent Technologies, RRID:SCR_013575) before loading on the Pacbio Sequel II system (PacBio Sequel II System, RRID:SCR_017990).

### DNA sequencing libraries construction and sequencing

For the first DNA sample, Illumina PE library preparation and sequencing were carried out by Macrogen, Inc. (Seoul, Korea), using Illumina HiSeq X Ten platform (Illumina HiSeq X Ten, RRID:SCR_016385), with 250 bp PE configuration. For the second specimen, PacBio HiFi library preparation and sequencing were performed at BYU, following the manufacturer’s recommendations [15]. The size-selected fraction had a mean read length of 15.3 kbp and was selected on the SageELF system (Sage Science, RRID:SCR_014808). The sequencing was conducted on two single-molecule, real-time (SMRT) cells using Sequel II system v.9.0, with a run time of 30 h, and 2.9 h pre-extension. The circular consensus analysis was performed in SMRT^®^ Link v9.0 [16] under default settings (the statistics of raw data generated from each PacBio SMRT cell can be viewed in Additional File 1 [17, 18]).

### Raw data quality control, clean-up, and genome size estimation

**Figure 2.**
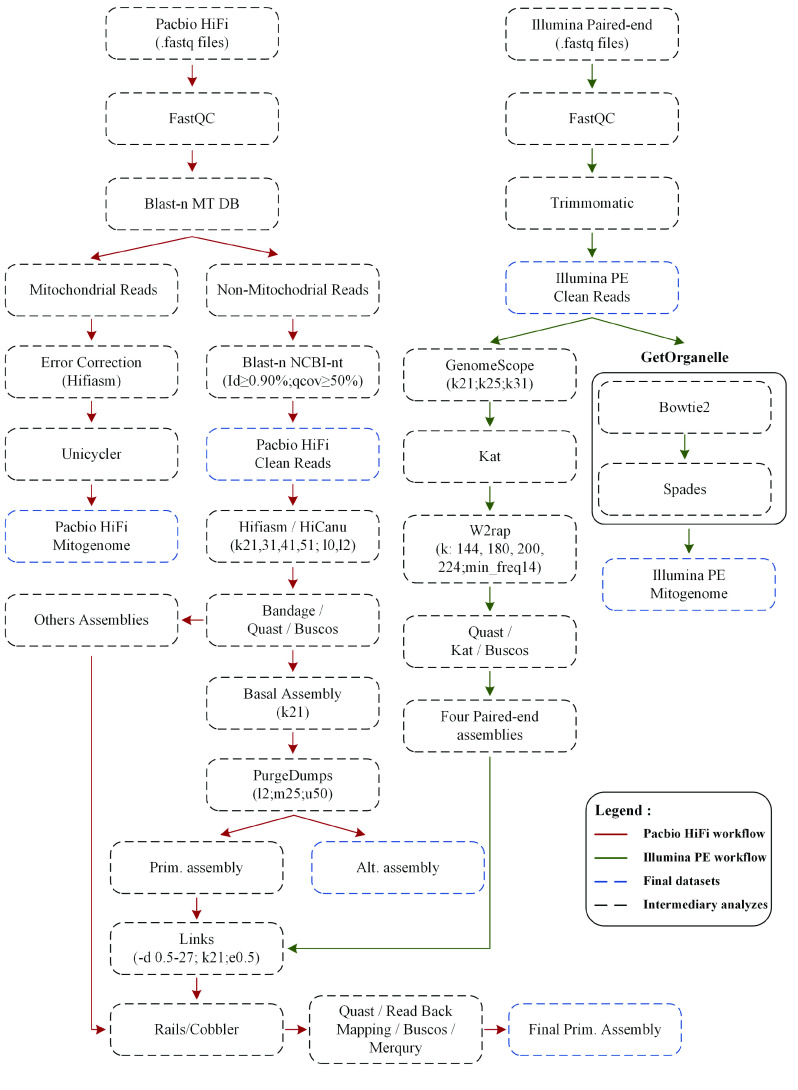
Bioinformatics workflow used to perform the genome assembly of *Scomber colias* species.

Both short- and long-read datasets were assessed by FastQC v.0.11.8 software (FastQC, RRID:SCR_014583). Trimmomatic v.0.38 software (Trimmomatic, RRID:SCR_011848) [19] was used to filter and remove low quality reads as well as the adaptors of the Illumina dataset (LEADING:5 TRAILING:5 SLIDINGWINDOW:4:20 MINLEN:50). Next, trimmed datasets were used to check the overall characteristics of the *S. colias* genome (i.e., genome size, heterozygosity, unique content), through GenomeScope 2.0 [20]. Briefly, Jellyfish v.2.2.10 software (Jellyfish, RRID:SCR_005491) [21] was used to build *k*-mer frequency distributions, and the final *k*-mer counts (*k*-mer 21, 25, 31) were submitted to the GenomeScope 2.0 online platform. On the other hand, HiFi reads were filtered in two ways (Figure 2). First, mitochondrial reads were removed by BLAST searches (BLASTN, RRID:SCR_001598) using a prebuilt database of mitochondrial sequences (database build protocol: (1) select all complete mitogenomes present in the nucleotide database of the National Center for Biotechnology Information (NT-NCBI); (2) select by taxon (Actinopterygii; txid:7898); (3) sequence length filter 15,000–50,000 bp; (4) build a database with the makeblastdb application of NCBI-BLAST+ v.2.9.0). Second, to filter out possible sources of contamination (artefactual or biological), HiFi reads were checked by BLAST (BLASTN) against NT-NCBI. Only HiFi reads with match hits over 90% identity and query coverage of 50% in the Actinopterygii taxon (NCBI:txid7898), or without match hits at all, were considered for further analysis (Figure 2).

### Mitochondrial genome assembly

Given that 2 specimens were used for the distinct sequencing approaches, i.e., PacBio HiFi and Illumina PE, the whole mitochondrial genome (mtDNA) was assembled and characterised for both specimens. For specimen 1, trimmed Illumina PE reads were used to assemble mtDNA in GetOrganelle v.1.7.1 [22] with optimised parameters (-F animal_mt -w 121 -R 10 -k 85,95,105,115,125) (Figure 2). For specimen 2, a new pipeline was designed to produce a mtDNA assembly from the PacBio HiFi long reads (Figure 2). The PacBio HiFi mtDNA reads, previously filtered (see above), were corrected using Hifiasm v.0.13-r308 (Hifiasm, RRID:SCR_021069) [23] with optimised parameters (–write-ec). Since Hifiasm is not optimised to assemble circular molecules (which are expected for mtDNA), the corrected PacBio HiFi mtDNA reads were assembled using Unicycler v.0.4.8 [24], a software package designed to assemble bacterial genomes and so optimised for circular assemblies, with default parameters. Annotation and visual representation of both mtDNA assemblies were produced using MitoZ v.2.3 [25] with optimised parameters (–genetic_code 2; –clade Chordata; –topology circular), using the PE reads for coverage plotting. Furthermore, annotations were manually validated by comparison with other mitochondrial genomes of the genus *Scomber*, available at NCBI (see Data Availability).

### Nuclear genome assembly and assessment

For whole-genome assembly a combined approach, using short- and long-read assemblies, was applied (Figure 2). While long-read assemblies were mainly used to produce the primary assembly, short-read assemblies were used to scaffold and improve the contiguity of the basal assembly. In summary, short-read assemblies were performed with the W2RAP pipeline v.0.1 [26], following the authors’ protocol. First the *k*-mer analyses toolkit (KAT) v.2.4.1 software (KAT, RRID:SCR_016741) [27] hist module was applied to determine the ideal *k*-mer cut-off, before W2RAP with optimised parameters (-t 30; -m 500; –min_freq 14; -d 32; –dump_all 1; -k: 144, 180, 200, 224) was used to produce 4 assemblies (Figure 2). To generate the long-read assembly, multiple software and parameters were initially tested. PacBio HiFi reads were assembled in Hifiasm v.0.13-r308 [23] with a range of parameters (*k* = 21, 25, 31, 41, 45, 51; *l* = 0, 2) and in HiCanu v.2.1.1 [28] with optimised parameters (default). While Hifiasm generated 2 pseudo-haplotypes per assembly, HiCanu generated 1 merged assembly. To choose the “best” assembly we applied a series of analyses, including Bandage (a bioinformatics application for nagivating *de novo* assembly graphs easily) v.0.8.1 [29] and manual inspection; Benchmarking Universal Single-Copy Orthologs (BUSCO) v.5.2.2 (BUSCO, RRID:SCR_015008) [30] with Eukaryota and Actinopterygii databases was used to assess the gene completeness of the assemblies, and Quality Assessment Tool for Genome Assemblies (QUAST) v.5.0.2 (QUAST, RRID:SCR_001228) [31], to check general metrics of the assemblies (Figure 2). Due to discrepancies in the length of the Hifiasm primary and alternative pseudo-haplotypes, we chose to concatenate them in a single assembly. At this point, the assembly with the highest complete BUSCO scores, highest contiguity (N50), and longest contig, was selected for further analysis. The pseudo-haplotypes were separated by purge_dups v.1.2.5 (purge dups, RRID:SCR_021173) [32]. After the first round of purging and inspection by *k*-mer plot, produced by the KAT tool, cutoffs were manually adjusted. To assess the influence of purge_dups in the genome, BUSCO (rate of deduplicates) and QUAST (N50 and genomic length per pseudo-haplotype) were used. Next, to improve the contiguity and quality of the assembly, short-read assemblies were used to structurally scaffold the assembly without the introduction of any new bases in the assembly, similar to the literature [33, 34] (Figure 2). The 4 short-read assemblies were inputted to the Long Interval Nucleotide *K*-mer Scaffolder (LINKS) v.1.8.7 [35], being used as long reads; using several distance values, i.e., -d 0.5, 1.5, 3, 9, 27 kb, the primary assembly was rescaffolded interactively for 5 rounds (additional parameters: -k 21 -e 0.5). Furthermore, the scaffolded genome and the long-read assemblies, initially produced by Hifiasm and HiCanu and discarded based on contiguity and completeness, were inputted to Cobbler v.0.6.1 [36] and RAILS v.1.5.1 [36] pipeline, with default parameters. This allowed gap filling of ambiguity regions (produced by short-read scaffolding), and further rescaffolding using long-read information. To evaluate the final assembly, several metrics and software were used. In addition to BUSCO and QUAST metrics, read back mapping of paired-end (PE) reads with Burrows-Wheeler Aligner (BWA) v.0.7.17-r1198 (BWA, RRID:SCR_010910) [37], long reads with Minimap2 v.2.17 (Minimap2, RRID:SCR_018550) [38] and RNA sequencing (RNA-Seq) with Hisat2 v.2.2.0 (HISAT2, RRID:SCR_015530) [39, 40], were also applied. To check consensus quality (QV) and *k*-mer completeness we used Merqury v.1.1 [41] (Figure 2).

### Repeat masking, gene prediction, and annotation

The repetitive elements of the genome were predicted and masked by RepeatMasker v.4.0.7 (RepeatMasker, RRID:SCR_012954) [42] using homologous comparisons and *ab initio* predictions. First, the *de novo* library of repetitive elements was created with the RepeatModeler v.2.0.1 (RepeatModeler, RRID:SCR_015027) [43]. Next, the *ab initio* library, as well as the Dfam_consensus-20170127 (Dfam, RRID:SCR_021168) [44] and RepBase-20181026 (Repbase, RRID:SCR_021169) [45], were used in RepeatMaker to softmask the *S. colias* genome assembly. The genome annotation was performed with the BRAKER2 pipeline v.2.1.6 (BRAKER, RRID:SCR_018964) [46–48]. Initially, the liver RNA-Seq reads (accession number: SRR6367407 [10]) were downloaded, mapped against the *S. colias* genome assembly using Hisat2 v.2.2.0 [39, 40] with default parameters, and converted to BAM and sorted files using Samtools v.1.9 (SAMTOOLS, RRID:SCR_002105)  [49]. Additionally, we collected 89 proteomes from NCBI RefSeq (RefSeq, RRID:SCR_003496) [50] and Ensembl (Ensembl, RRID:SCR_002344) [51] databases. The species and accession numbers of the proteomes used in the genome annotation of *S. colias* can be consulted in Additional File 2 [17, 18]. Of these, 82 species belong to the class Actinopterygii (32 taxonomic orders): 81 with genome assembly at chromosome level, and 1 at scaffold level. As of the date of this genome annotation, only 1 Scombriforme genome, *Thunnus orientalis*, was annotated at scaffold level. The remaining 7 proteomes were selected from other vertebrate non-teleost animal models: *Callorhinchus milii*, *Amblyraja radiata*, *Scyliorhinus canicula*, *Lepisosteus oculatus*, *Petromyzon marinus*, *Mus musculus*, and *Homo sapiens*. Next, the RNA-Seq alignment, as well as all the above-mentioned proteomes, were inputted to the BRAKER2 pipeline with optimised parameters (–etpmode; –softmasking; –UTR = off; –crf; –cores = 30). The final file of predictions (braker.gtf) was further filtered by evidence, keeping only gene predictions with RNA-Seq or protein evidence (using BRAKER2 auxiliary scripts; selectSupportedSubsets.py), then converted to .gff3 format (using the Augustus auxiliary scripts; gtf2gff.pl) and post-processed with Another Gtf/Gff Analysis Toolkit (AGAT) v.0.6.0 [52]. The post-processing stage involved the correction of overlapping gene prediction coordinates and the removal of small or incomplete protein-coding genes (i.e., coding for <100 amino acids (aa); lacking start or stop codons). Furthermore, the proteins were extracted with AGAT and subject to functional annotation using InterProScan v.5.44.80 (InterProScan, RRID:SCR_005829) [53] and BLASTP (BLASTP, RRID:SCR_001010) searches against RefSeq  [50] and UniProtKB/SwissProt (UniProtKB, RRID:SCR_004426) [54] databases. The homology searches were performed with DIAMOND v.2.0.11.149 (DIAMOND, RRID:SCR_016071) [55] with optimised parameters (-k 1, -b 10, -e 1e-5, –ultra-sensitive, –outfmt 6). Finally, the genome and the annotation datasets were integrated using JBrowse2 (JBrowse, RRID:SCR_001004) [56], a dynamic web platform for genome visualisation and analysis that allows easy and interactive exploration of provided data (http://portugalfishomics.ciimar.up.pt/app/scombercolias/). The FASTA file containing the genome was indexed with Samtools faidx v.1.9 [49] and added to the JBrowse component, along with the annotation file sorted with GenomeTools v.1.6.1 (GenomeTools, RRID:SCR_016120) [57], and indexed with Samtools tabix v.1.9 [58]. In addition to the JBrowse component, NCBI-BLAST+ v.2.12.0 [59] was integrated into the webpage, allowing BLAST results from the genome, mRNA, protein-coding sequences (CDS), and proteins, directly from the website.

### Phylogenomics

To generate a phylogenomic analysis, the proteomes of 15 selected Actinopterygii species, including the Scombriformes species *Thunnus maccoyii* and *T. orientalis*, were downloaded from public databases. The species and accession numbers used in the phylogenomic analyses can be consulted in Additional File 2 [17, 18]. Single-copy orthologs between these 15 species and *S. colias* were retrieved from the protein datasets by constructing protein family clusters using OrthoFinder v.2.4.0 (OrthoFinder, RRID:SCR_017118) [60] with optimised parameters (-M). This resulted in a total of 392 single-copy orthologous sequences that were individually aligned using MUSCLE v.3.8.31 (MUSCLE, RRID:SCR_011812) [61] with default parameters. Each alignment was trimmed using TrimAl v.1.2 (trimAl, RRID:SCR_017334) [62] with a gap threshold of 0.5 with optimised parameters (-gt 0.5), and afterwards concatenated using FASconCAT-G [63]. Phylogenetic inferences were conducted in IQ-Tree v.1.6.12 (IQ-Tree, RRID:SCR_017254) [64] with optimised parameters (-bb 10000 -nt AUTO -st AA). The best-fit molecular evolutionary model used in the phylogenetic analyses was JTT+F+R4, which was selected by ModelFinder [65] implemented within IQ-Tree.

### Assessing the nuclear receptor and the “*chemical defensome*” repertoire in *Scomber colias*

To demonstrate the value of the present genome resourse, we collected the repertoire of nuclear receptors (NRs) in *S. colias* via TBLASTN (TBLASTN, RRID:SCR_011822) searches in the primary genome assembly with default parameters. Protein sequences of DNA-binding domains and ligand-binding domains in *H. sapiens* NRs were collected from the RefSeq [50] database and used in a query (NP_000466.2, NP_068804.1, NP_003241.2, XP_005257609.1, NP_001349802.1, NP_068370.1, NP_599022.1, NP_009052.4, NP_001351014.1, XP_005260464.1, NP_002948.1, NP_001257330.1, NP_003288.2, XP_016862607.1, NP_001273031.1, NP_005645.1, NP_001278159.1, NP_004442.3, NP_000167.1, XP_005268879.1, NP_004950.2, NP_201591.2). Next, regions aligning with *H. sapiens* sequences were collected, translated to protein using the Bio.Seq module of Biopython v.1.75 (Biopython, RRID:SCR_007173) [66], and blasted (BLASTP) against a local database containing the NR proteins of *Danio rerio* (*D. rerio* NRs database protocol: (1) NRs sequences and classifications were retrieved from [67]; (2) an NRs database was built using the makeblastdb application of NCBI-BLAST+ v.2.12.0). For each NRs sequence in *S. colias*, the best blast hit in the *D. rerio* database was collected. In some cases, several NRs of *S. colias* matched the same receptor in *D. rerio*. In these cases, the nucleotide sequences of *S. colias* were again validated against the NT-NCBI database, and all sequences matching different GeneIDs in the same organism were kept in the final table of NRs. In parallel, and to assess the genome annotation performed by BRAKER2, the genomic coordinates of regions aligning with *H. sapiens* were searched and identified in the annotation files.

To identify the genes related to the *chemical defensome*, target genes were selected based on a previous report profiling the “chemical defensome” of teleost species [68]. Next, gene names were used as queries to search the deduced *S. colias* genome annotation, a simple but successful approach for well-annotated genomes such as *D. rerio* [68]. When gene names were not retrieved from *S. colias* genome annotation (i.e., *fthl*, *gstp*, *hsph*, *maff*, *nme8*, *slc21*), further TBLASTN searches were performed in the primary genome assembly with optimised parameters (-max_hsps 1 to keep the best query-subject pair), using *D. rerio* sequences as a query.

### Demography with pairwise sequentially Markovian coalescent (PSMC)

To explore the variation in the demographic history of the species, a pairwise sequentially Markovian coalescent (PSMC) (PSMC, RRID:SCR_017229) strategy was applied [69], following the authors’ instructions. Briefly, PE short reads were aligned to the repeated masked genome assembly using BWA v.0.7.17-r1198 (BWA, RRID:SCR_010910) [37] with optimised parameters (BWA-MEM), and the output converted to BAM format and sorted using Samtools v.1.9 [49] (function: sort; parameters: default). Next, Picard Tools v.2.19.2 (Picard, RRID:SCR_006525) was used to remove duplicate reads (function: MarkDuplicates; parameters: default), and SAMtools for mapping quality filtering and SNP calling (function: mpileup; parameters: -Q 30 -q 30 -C 50). BCFtools v.1.9 (SAMtools/BCFtools, RRID:SCR_005227) was applied to extract consensus sequences (function: call; parameters: -c), and the subscript vcfutils (from SAMtools) was used for filtering the output for a minimum depth of 25, a maximum depth of 150, and a min RMS mapQ of 20 (function: vcf2fq; parameters: -d 25 -D 150 -Q 20). The resulting fastq file was converted to a PSMC-compatible input format using fq2psmcfa with a minimum quality threshold of 20 (parameters: -q 20). Inferences of population history were performed by running PSMC for 25 iterations with optimised parameters (-N 15, -r 5, -p 4*4 + 13*2 + 4*4 + 6) following recent PSCM estimations on Scombriformes [70]. Furthermore, to account for uncertainties in the PSMC estimates, bootstrapping of 100 replicates was performed using the split face script provided by PSMC authors. Finally, to scale the demographic estimations, a mutation rate (μ) of 7.3 × 10^−9^ substitutions/site/generation was used, based on a recent estimation for the Scombriformes species *Thunnus albacares* [70], and a generation time for *S. colias* of 2 years [7, 71].

## Data validation

To produce the *S. colias* genome assembly, 2 sequencing strategies were used: Illumina PE short reads and PacBio HiFi long reads. The PE dataset was used to assess the genomic proprieties of the *S. colias* species and scaffold the long-read assembly, while HiFi reads were used to perform the primary genome assembly and gap closing (Figure 2).

**Figure 3.**
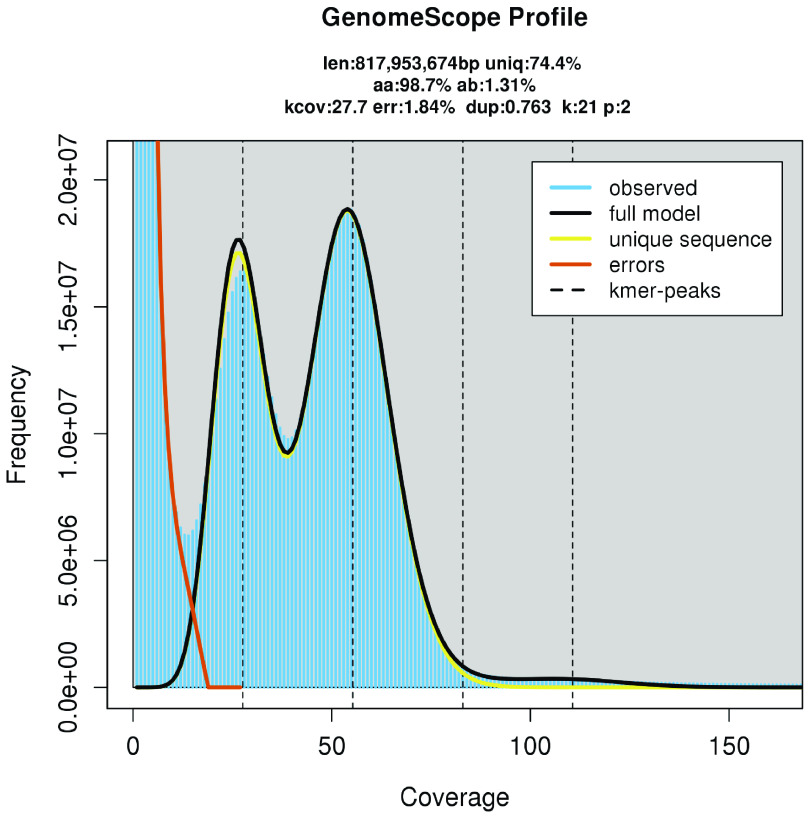
Genomescope2 plot with *k*-mer spectra content and fitted models of the *Scomber colias* Illumina PE dataset.

The Illumina sequencing yielded 149 M of PE reads and the PacBio sequencing generated 1.7 M of HiFi reads (Table 1). Trimmed short reads were used to estimate the genome size (817 Mbp), heterozygosity rate (1.31%), and genome repeat content (approximately 26%), using GenomeScope2 (Figure 3). The complete statistics of GenomeScope2 can be consulted in Additional File 3 [17, 18]. In parallel, the HiFi dataset was inspected, and mitochondrial reads, as well as possible sources of contamination, were removed (amounting to 0.31% of the initial dataset) (Table 1).

**Figure 4.**
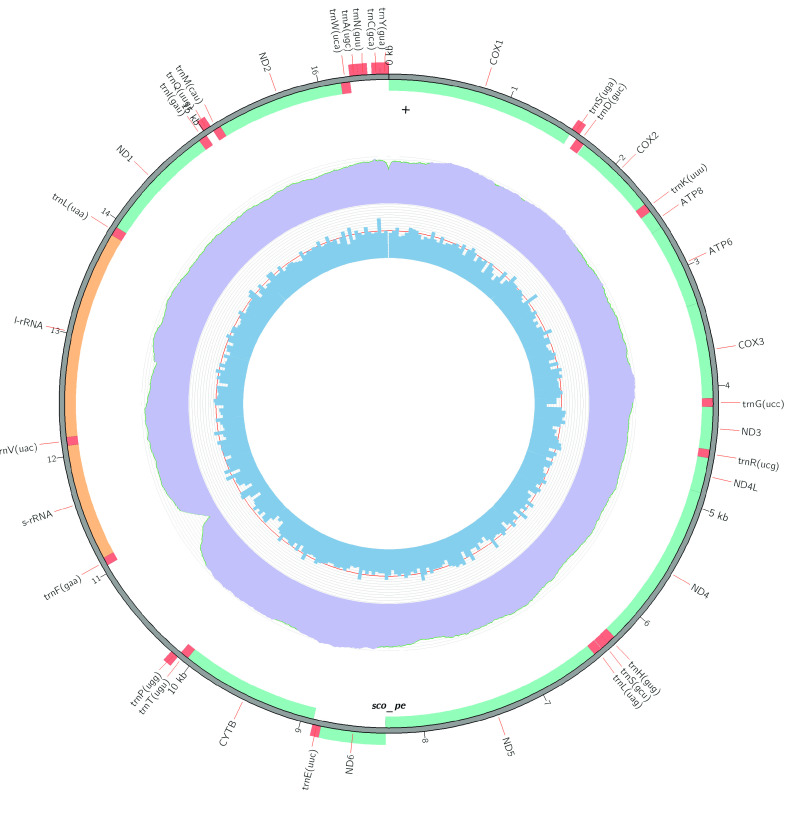
Circular mitochondrial genome assembly of *Scomber colias*, obtained from the Illumina PE dataset (equal to that obtained from the PacBio HiFi long reads assembly). From the centre to the outmost features: GC content distribution; sequencing depth distribution of aligned Paired-End reads; gene elements (i.e., PCGs, rRNA genes, tRNA genes).

For the mtDNA assemblies, a total of 38,868 mtDNA PE reads were filtered by GetOrganelle and a total of 792 mtDNA PacBio HiFi reads were filtered by BLASTN search. The 2 assemblies had the same length, 16,570 bp, and differed from each other by 0.29% (uncorrected *p*-distances). Furthermore, the PE and PacBio HiFi mtDNA assemblies differed from the *S. colias* mtDNA assembly available on NCBI (accession number AB488406.1 [11]), by 0.35% and 0.40% respectively (uncorrected *p*-distances). The mtDNA gene content and arrangement is as expected for most fishes and is standard for vertebrates [72], consisting of 13 protein-coding genes, 22 transfer RNA (trn), and 2 ribosomal RNA (rrn) (Figure 4).

**Table 1:** General statistics of read datasets used to perform the *Scomber colias* genome assembly.

Sample	Sequencing type	Library type	Platform	Insert size (bp)	Number of reads (before clean-up)	Number of reads (after clean-up)	Application
Sco_PH	WGS	Long reads	PacBio Sequel II System	15,500	1,792,104	1,786,541	Genome Assembly, Gap Closing, Assessment
Sco_PE	WGS	Short reads	HiSeq X Ten	478	149,564,893	84,738,393	Scaffold, Assessment

**Figure 5.**
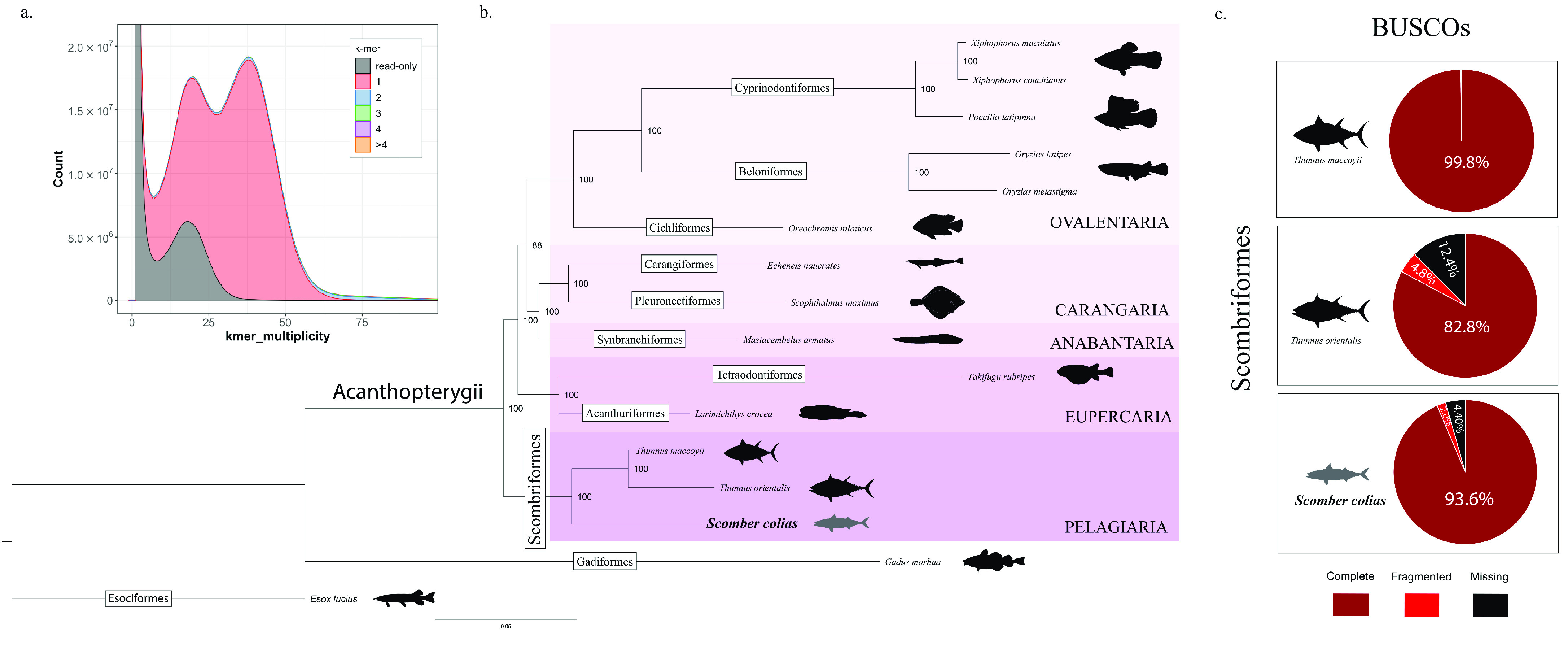
Validation of the genome assembly and annotation process. (a) *K*-mer analyses of the *Scomber colias* genome assembly (Merqury). (b) Maximum Likelihood phylogenetic tree based on the concatenated alignments of amino acid sequences of 392 single-copy orthologs retrieved by OrthoFinder. Bootstrap values are shown next to the nodes. (c) BUSCO scores were obtained from searching the proteomes of the 3 Scombriformes species with genome annotation available, against the actinopterygii_odb10 (n:3640) lineage.

The primary genome assembly was produced using filtered PacBio HiFi reads and the below software packages and settings. Following the above-mentioned criteria (Material and Methods: Nuclear genome assembly and assessment) the Sco_k21 assembly was selected, with both pseudo-haplotypes merged and subjected to purge_dups. Detailed statistics of Hifiasm and HiCanu genome assemblies can be consulted in Additional File 4 [17, 18]. Although the purge_dumps generated a primary and an alternative assembly, only the primary assembly was used in subsequent steps. At the same time, 4 short-read genome assemblies were performed with W2RAP software, and contigs with over 500 bp were used as “long reads” to scaffold the primary assembly. Additional File 5 shows QUAST and BUSCO statistics for the PE genome assemblies [17, 18]. Importantly, during the scaffolding process, only structural information of short-read assemblies was used, without the inclusion of bases. Lastly, the remaining non-basal long read assemblies were used to fill gaps inserted during the scaffolding stage. The final assembly (primary assembly) of *S. Colias* yielded a genome size of 814 Mbp, distributed in 2,028 scaffolds and 2,093 contigs with an N50 length of 4.19 and 3.34 Mbp, respectively. On the other hand, the alternative assembly had 807 Mbp and 5908 contigs with an N50 length of 0.47 Mbp (Table 2). The BUSCO analyses, at the nucleotide level, in Eukaryota and Actinopterygii datasets, showed high levels of completeness for both primary (97.3% and 97.9% of single-copy orthologs) and alternative (93.3% and 96% single-copy orthologs) assemblies (Table 2). Consistently, Merqury determined high QV (primary, 56.53%; alternative, 54.99%) and *k*-mer completeness (primary, 86.11%; alternative, 84.60%) values for both assemblies (Table 2). In the primary assembly, the *k*-mer analyses (via Merqury) showed a low level of *k*-mer duplication in the genome (colour blue, green, purple, and orange in Figure 5a), indicating a high level of haplotype uniqueness (red colouring in Figure 5a), and a similar *k*-mer distribution pattern to GenomeScope2 (performed with Illumina PE reads). Additionally, we found a high mapping rate in the Illumina, PacBio, and RNA-Seq reads, against the primary assembly of 95%, 99.8%, and 90.02%, respectively. Overall, these results provide evidence of the high quality of the *S. colias* genome assembly (Table 2). Our *S. colias* genome assembly ranks fourth in high-quality genome assemblies within the order Scombriformes and first in the genus *Scomber* (Additional File 6) [17, 18].

**Table 2:** Statistics of the *Scomber colias* genome assembly.

Assembly	Alternative	Primary	
	Contigs (Sco_k21_a_c)	Contigs (**Sco_k21_p_c)**	Scaffolds (Sco_k21_p_s)
Number of contigs (≥10,000 bp)	5,908	2,093	2,028
Number of contigs (≥50,000 bp)	2,417	1,123	1,078
Number of contigs (≥100,000 bp)	1,593	704	662
Number of contigs (≥200,000 bp)	1,025	456	417
Number of contigs (≥500,000 bp)	421	235	209
Number of contigs (≥1,000,000 bp)	123	155	138
Total length (≥10,000 bp)	807,928,680	813,976,802	814,072,661
Total length (≥50,000 bp)	721,244,010	781,696,683	782,480,923
Total length (≥100,000 bp)	662,374,873	751,893,146	752,912,084
Total length (≥200,000 bp)	580,469,606	716,806,065	718,068,371
Total length (≥500,000 bp)	385,329,197	648,055,626	653,890,381
Total length (≥1,000,000 bp)	180,689,595	591,655,104	603,146,189
Largest contig (Mbp)	3,248	22,804,600	22,804,600
Total length (Mbp)	807,936	813,977	814,072
GC (%)	39.94	40.09	40.09
N50 (Mbp)	0,466	3,342	4,190
*K*-mer completeness (%)	84.602		86.1077
Consensus quality	56.5369		54.9969
Read back mapping PE (%)	-		95.0
Read back mapping PH (%)	-		99.8
Read back mapping RNA-Seq (%)	-		90.2
**BUSCO statistics (databases)**	-		
Eukariota**	T: 93.3, C: 90.2 [S: 88.6, D: 1.6], F: 3.1, M: 6.7, n: 255	T: 97.3, C: 96.1 [S: 93.7, D: 2.4], F: 1.2, M: 2.7, n: 255	
Actinopterygii**	T: 96.0, C: 94.8 [S: 91.9, D: 2.9], F: 1.2, M: 4.0, n: 3640	T: 97.9, C: 97.2 [S: 96.2, D: 1.0], F: 0.7, M: 2.1, n: 3640	

* Statistics are based on contigs and scaffolds of size ≥1000 bp. ** (T, total BUSCOs found (completed + fragmented), %; C, complete BUSCOs [S, complete and single copy, %; D, complete and duplicated, %]; F, fragmented, %; M, missing, %; n, number of sequences in database).

The RepeatMasker software masked 29.62% of bases in the primary genome assembly. The masked regions were predominantly linked to DNA elements (11.66%), long interspersed nuclear elements (4.11%), long terminal repeats (2.58%), and simple repeats (2.88%). Furthermore, 8.62% of the genome was masked and annotated as “unclassified”, and only a small percentage were classified as short interspersed nuclear elements, small RNA, or satellite repeats (Table 3). The genome annotation process generated about 27,675 protein-coding genes and 30,999 protein-coding sequences. On average, we found 9.5 exons and 1,656 bp lengths per CDS (Table 4). Of the CDS, 30,355 had at least 1 BLASTP hit in SwissProt or RefSeq databases, 27,101 were identified in the InterPro database, and 21,664 of these were classified as belonging to a specific homolog superfamily (Table 5).

**Table 3:** Report of RepeatMasker software. This report contains statistics of repetitive elements in the *Scomber colias* genome assembly.

Total number of sequences	2,028		
Total length (bp)	814,072,661 bp		
GC level (%)	40.09		
Number of bases masked	241,071,029 bp (29.62%)		
Type	Number of elements	Length in Genome	Percentage of Genome
**SINEs**	16,132	2,679,916	0.33
ALUs	0	0	0.00
MIRs	7,082	1,280,739	0.16
**LINEs**	113,089	33,426,533	4.11
LINE1	8,048	4,651,362	0.57
LINE2	57,670	14,551,177	1.79
L3/CR1	697	123,438	0.02
**LTR elements**	82,410	20,969,171	2.58
ERVL	10	279	0.00
ERVL-MaLRs	0	0	0.00
ERV_classI	22,786	4,702,084	0.58
ERV_classII 11490		576,448	0.07
**DNA elements**	623,126	94,930,706	11.66
hAT-Charlie	27,952	5,526,534	0.68
TcMar-Tigger	169	46,619	0.01
**Unclassified**	278,199	70,161,089	8.62
**Total interspersed repeats**	-	222,167,415	27.29
**Small RNA**	11,380	1,807,250	0.22
**Satellites**	18,552	3,093,792	0.38
**Simple repeats**	84,014	23,465,814	2.88
**Low complexity**	959	200,769	0.02

**Table 4:** Structural annotation report of the *Scomber colias* genome assembly.

Structural Annotation	Result
Number of genes	27,675
Number of mRNAs	30,999
Number of CDSs	30,999
Number of exons	295,102
Number of introns	264,103
Number of exon in CDS	295,102
Number of intron in CDS	264,103
Number of introns in exon	264,103
Number of introns in intron	235,209
Number gene overlapping	71
Number of single exon genes	2,036
Number of single exon mRNA	2,105
Mean mRNAs per gene	1.1
Mean CDSs per mRNA	1.0
Mean exons per mRNA	9.5
Mean introns per mRNA	8.5
Mean exons per CDS	9.5
Mean introns in CDSs per mRNA	8.5
Mean introns in exons per mRNA	8.5
Mean introns in introns per mRNA	7.6
Total gene length	269,856,447
Total mRNA length	310,580,471
Total CDS length	51,346,678
Total exon length	51,346,678
Total intron length	259,233,793
Total intron length per CDS	259,233,793
Total intron length per exon	259,233,793
Total intron length per intron	35,919,947
Mean gene length	9,750
Mean mRNA length	10,019
Mean CDS length	1,656
Mean exon length	173
Mean intron length	981
Mean intron in exon length	981
Mean intron in intron length	152
Longest gene	242,447
Longest mRNA	242,447
Longest CDS	98,436
Longest exon	14,939
Longest intron	76,003
Longest CDS piece	14,939
Shortest gene	303
Shortest mRNA	144
Shortest CDS	18
Shortest exon	3
Shortest intron	30

**Table 5:** Functional annotation report of *S. colias* genome assembly.

Functional Annotation	Number
Swiss-Prot/ RefSeq	30,355
InterPro	27,101
CDD	12,832
Coils	7,705
GO	18,643
Gene3D	22,209
HAMAP	463
KEGG	1,402
MetaCyc	1,140
MobiDBlite	16,765
PIRSF	1,755
PRINTS	7,143
Pfam	25,708
PROSITE patterns	8,082
PROSITE profiles	16,229
Reactome	7,376
SFDL	114
SMART	14,906
SUPERFAMILY	21,664
TIGRFAMs	1,427

To validate the protein-coding sequences we performed phylogenetic analysis (via OrthoFinder) and BUSCO analysis (using the Actinopterygii library profile) (Figure 5b, c). Of the 16 Actinopterygii proteins datasets inputted to OrthoFinder, 98.3% were assigned to 29,066 orthogroups, with 12,334 orthogroups present in all species. All OrthoFinder statistics can be consulted in Additional File 7 [17, 18]. Furthermore, a total of 392 single-copy orthologues were retrieved by OrthoFinder and used for the phylogenomic analysis. Alignment, trimming and concatenation of all single-copy orthologues, resulted in a final 120,886 aa-long supermatrix alignment that was used for phylogenomic inference in IQ-Tree. The resulting Maximum Likelihood phylogenetic tree has maximum support for almost all nodes (Figure 5b). The phylogeny recovered the reciprocal monophyletic Acanthopterygii groups Pelagiaria, Eupercaria, Anabantaria, Carangaria, and Ovalentaria, with Pelagiaria being the basal clade and represented by the 3 Scombrifomes, including *S. colias* (Figure 5b). These results are in accordance with the most recent phylogenomic study of ray-finned fishes [73], as well as the Ensembl Compara Species Tree of Ensembl database  [51]. BUSCO analysis showed the *S. colias* proteome with 93.6% of the groups complete, 2% fragmented, and 4.4% missing (Figure 5c). In comparison, *T. maccoyii* had 99.8% BUSCO groups complete, while *T. orientalis* had but 82.8%. These results are expected, since the *T. maccoyii* genome assembly, part of the Vertebrate Genome Project [74], was built at chromosome level, with multiple technologies (including 46x PacBio data, 46x 10X Genomics Chromium data, BioNano data, and Arima Hi-C data) and several manual curation steps  [75]. In contrast, both *T. Orientalis* [76] and *S. colias* were built at scaffold level using only short- and long-read information.

We further explored the quality of the annotation by investigating the repertoire of the NRs superfamily in the *S. colias* assembly. NRs are critical molecular physiology components, with vital roles in animal physiology and disruption [77]. Moreover, their exact NR gene complement in vertebrate lineages has been shown to vary  [67]. We were able to deduce the existence of 76 NRs in the *S. colias* genome, detailed in Additional File 8, in line with the repertoire described for other teleost species [78]. Among the retrieved NRs we found those that are key components of the “chemical defensome”—an ensemble of related and unrelated genes that protect organisms against chemical stressors, and are thus critical under anthropogenic chemical build-up and climate change scenarios—such as the xenobiotic-inducible pregnane X receptor (*pxr*, *nr1i2*) [68, 79]. Subsequent analysis, using gene names, further suggested the presence of gene annotations for the vast majority of the reported members of the teleost “chemical defensome” in *S. colias*, similarly to that described for *D. rerio* [68]. Additional BLAST searches were performed for a reduced set of genes (*fthl*, *gstp*, *hsph*, *maff*, *nme8,* and *slc21*), uncovering possible homologs for this gene subset, except for a single member of the GST family (*gstp*). The chemical defensome repertoire identified in *S. colias* species is detailed in Additional File 9 in the associated data entries [17, 18].

**Figure 6.**
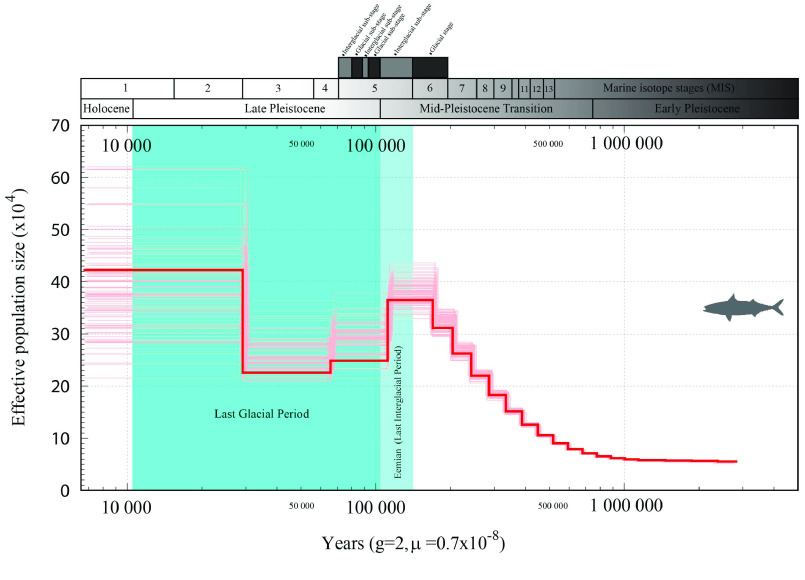
Pairwise sequentially Markovian coalescent (PSMC) estimates from the *Scomber colias* genome assembly. Estimations were obtained using a generation time of 2 years and genome-wide mutation rate of 7.3 × 10^−9^ mutations/site/generation. Effective population size (*N*_*e*_) is presented in the left vertical axis, and changes estimated up to the present, over the last 3 myr, on the horizontal axis.

We additionally validated our dataset by examining the present population structure of the species, since the genome may also provide clues regarding its past demographic history [69]. One popular method to produce these inferences is the pairwise sequentially Markovian coalescent (PSMC) model, here applied to the *S. colias* final genome assembly. Since PSMC requires an estimation of the genome-wide mutation rate, and since this has never previously been produced for *S. colias*, we used the recently estimated genome-wide mutation rate of the yellowfin tuna, *T. albacares*, of 7.3 × 10^−9^ mutations/site/generation [70]. The results suggest a past population expansion between 160,000–115,000 years ago, with maximum effective population size (*N*_*e*_) of 36,000 during the end of the Mid-Pleistocene Transaction, corresponding to the Eemian (i.e., the last interglacial period) and the transition between Marine Isotope Stages (MIS) 5 and 6 (Figure 6). This population expansion is followed by an apparent decrease in the *N*_*e*_ to around 25,000 at the beginning of the Late Pleistocene, corresponding to the beginning of the Last Glacial Period. These results, suggesting the influence of climatic changes from the Pleistocene glaciation cycles on the *N*_*e*_, are following other recent studies on Scombriformes, such as the Pacific Sierra mackerel, *Scomberomorus sierra* [80], and the Indo-Pacific Yellowfin tuna *T. albacares* [70], as well as in other pelagic marine species such as the killer whale [81].

## Reuse potential

This study reports the first genome assembly of Atlantic chub mackerel. *Scomber colias* is a valuable marine resource, with a high impact on the fisheries of several countries along the west coast of the Atlantic Ocean and the Mediterranean Sea. Ecologically, this species establishes an important link between primary producers and top predators of the marine trophic web. Despite the ecological and economic importance of *S. colias*, few genomic resources are available for this species. Thus, this genome is timely and is expected to contribute to the effective conservation, management, and sustainable exploitation of *S. colias* species in the Anthropocene. Additionally, this genome will be a key tool to decipher biological features of *S. colias*, such as population dynamics, ecology, and physiology.

## Data Availability

Raw datasets of PacBio HiFi and Illumina sequencing were deposited in the NCBI Sequences Read Archive under Bioproject PRJNA769550. Additionally, both primary and alternative pseudo-haplotype assemblies were submitted to NCBI GenBank (accession numbers JAJDFG000000000 and JAJDFH000000000). Mitochondrial genome assemblies and annotations were submitted to GenBank (accession numbers OK501306 and OK501307). The four W2RAP assemblies, as well as genome annotation files and supplementary tables, were uploaded to Figshare online repository [17]. Additional data is available at the *GigaScience* GigaDB repository [18]. Genome and annotation datasets also can be interactively explored at http://portugalfishomics.ciimar.up.pt/app/scombercolias/.

## List of abbreviations

aa: amino acid; AGAT: Another Gtf/Gff Analysis Toolkit; bp: base pair(s); BUSCO: Benchmarking Universal Single-Copy Ortholog; BWA: Burrows-Wheeler Aligner; CDS: protein-coding sequences; DHA: docosahexaenoic acid; gDNA: genomic DNA; HiFi: High-Fidelity; KAT: *k*-mer analyses toolkit; LINKS: long interval nucleotide *k*-mer scaffolder; Mbp: megabase pair(s); mtDNA: mitochondrial genome; NCBI: National Center for Biotechnology Information; NRs: nuclear receptors; NT-NCBI: nucleotide database of NCBI; PacBio: Pacific Biosciences; PE: paired-end; PSMC: pairwise sequentially Markovian coalescent; QUAST: Quality Assessment Tool for Genome Assemblies; QV: consensus quality value; RNA-Seq: RNA sequencing; SMRT: single molecule, real-time; SNP: single-nucleotide polymorphism.

## References

[1] Collette BB, Nauer CE, Scombrids of the world. An Annotated and Illustrated Catalogue of Tunas, Mackerels, Bonitos and Related Species Known to Date. FAO Species Cat., 1983; 2: 2–137. http://www.fao.org/3/ac478e/ac478e00.htm. Accessed 20 Jan 2020.

[2] Hernández JJC, Ortega ATS, Castro Hernandez JJ Synopsis of biological data on the chub mackerel (*Scomber japonicus* Houttuyn, 1782.. FAO Fish Synop., 2000; 157: 1–77. https://agris.fao.org/agris-search/search.do?recordID=XF2000393177. Accessed 20 Feb 2020.

[3] Velasco EM, del Arbol J, Baro J Age and growth of the Spanish chub mackerel *Scomber colias* off southern Spain: a comparison between samples from the NE Atlantic and the SW Mediterranean. Rev. Biol. Mar. Oceanogr., 2011; 46: 27–34. doi:10.4067/S0718-19572011000100004.

[4] Gamito R, Pita C, Teixeira C Trends in landings and vulnerability to climate change in different fleet components in the Portuguese coast. Fish Res., 2016; 181: 93–101. doi:10.1016/j.fishres.2016.04.008.

[5] Karakoltsidis PA, Zotos A, Constantinides SM, Composition of the commercially important mediterranean finfish, crustaceans, and molluscs. J. Food Compos. Anal., 1995; 8: 258–273. doi:10.1006/jfca.1995.1019.

[6] Ferreira I, Gomes-Bispo A, Lourenço H The chemical composition and lipid profile of the chub mackerel (*Scomber colias*) show a strong seasonal dependence: Contribution to a nutritional evaluation. Biochimie, 2020; 178: 181–189. doi:10.1016/j.biochi.2020.09.022. 32980464

[7] Carvalho N, Perrotta RG, Isidro E, Age, growth and maturity in the chub mackerel (*Scomber japonicus* Houttuyn, 1782) from the Azores. Arquipél. Ciênc. Biol. Mar., 2002; 19: 93–99. https://repositorio.uac.pt/bitstream/10400.3/169/1/pp93_99_Carvalho_et_al.pdf.

[8] Martins MM, Skagen D, Marques V Changes in the abundance and spatial distribution of the Atlantic chub mackerel (*Scomber colias*) in the pelagic ecosystem and fisheries off Portugal. Sci. Mar., 2013; 77: 551–563. doi:10.3989/scimar.03861.07B.

[9] Vasconcelos J, Afonso-Dias M, Faria G, Atlantic chub mackerel (*Scomber colias*) spawning season, size and age at first maturity in Madeira waters. Arquipelago Life Mar. Sci., 2012; 29: 43–51. https://www.researchgate.net/publication/273319854.

[10] Machado AM, Felício M, Fonseca E A resource for sustainable management: De novo assembly and annotation of the liver transcriptome of the Atlantic chub mackerel, *Scomber colias*. Data Br., 2018; 18: 276–284. doi:10.1016/j.dib.2018.03.013. PMC5996228 29896516

[11] Catanese G, Manchado M, Infante C, Evolutionary relatedness of mackerels of the genus Scomber based on complete mitochondrial genomes: Strong support to the recognition of Atlantic Scomber colias and Pacific Scomber japonicus as distinct species. Gene, 2010; 452: 35–43. doi:10.1016/j.gene.2009.12.004. 20035845

[12] Rodríguez-Ezpeleta N, Bradbury IR, Mendibil I Population structure of Atlantic mackerel inferred from RAD-seq-derived SNP markers: effects of sequence clustering parameters and hierarchical SNP selection. Mol. Ecol. Resour., 2016; 16: 991–1001. doi:10.1111/1755-0998.12518. 26936210

[13] Ravi V, Venkatesh B, The divergent genomes of teleosts. Annu. Rev. Anim. Biosci., 2018; 6: 47–68. doi:10.1146/annurev-animal-030117-014821. 29447475

[14] Formenti G, Theissinger K, Fernandes C The era of reference genomes in conservation genomics. Trends Ecol. Evol. 2022; 37(3): 197–202. doi:10.1016/j.tree.2021.11.008. 35086739 PMC13065249

[15] PACBIO. Procedure & Checklist – Preparing HiFi SMRTbell^®^ Libraries using the SMRTbell Express Template Prep Kit 2.0. Pacific Biosciences, CA; 2021; https://www.pacb.com/wp-content/uploads/Procedure-Checklist-Preparing-HiFi-SMRTbell-Libraries-using-SMRTbell-Express-Template-Prep-Kit-2.0.pdf.

[16] PACBIO. SMRT link software installation (v9.0). Pacific Biosciences, CA; 2020; https://www.pacb.com/wp-content/uploads/SMRT_Link_Installation_v90.pdf.

[17] Machado A, A draft genome assembly of the Atlantic chub mackerel (*Scomber colias*) using Illumina and Pacbio Hifi reads. Figshare Dataset. 2021; 10.6084/m9.Figshare.17025506.v4.

[18] Machado AM, Gomes-dos-Santos A, Fonseca MM Supporting data for “A genome assembly of the Atlantic chub mackerel (*Scomber colias*): a valuable teleost fishing resource”. GigaScience Database. 2022; 10.5524/100978. PMC9650269 36824513

[19] Bolger AM, Lohse M, Usadel B, Trimmomatic: A flexible trimmer for Illumina sequence data. Bioinformatics, 2014; 30: 2114–2120. doi:10.1093/bioinformatics/btu170. 24695404 PMC4103590

[20] Ranallo-Benavidez TR, Jaron KS, Schatz MC, GenomeScope 2.0 and Smudgeplot for reference-free profiling of polyploid genomes. Nat. Commun., 2020; 11: 1–10. doi:10.1038/s41467-020-14998-3. 32188846 PMC7080791

[21] Marçais G, Kingsford C, A fast, lock-free approach for efficient parallel counting of occurrences of *k*-mers. Bioinformatics, 2011; 27: 764–770. doi:10.1093/bioinformatics/btr011. 21217122 PMC3051319

[22] Jin JJ, Yu W Bin, Yang JB GetOrganelle: A fast and versatile toolkit for accurate de novo assembly of organelle genomes. Genome Biol., 2020; 21: 241. doi:10.1186/s13059-020-02154-5. 32912315 PMC7488116

[23] Cheng H, Concepcion GT, Feng X Haplotype-resolved de novo assembly using phased assembly graphs with hifiasm. Nat. Methods, 2021; 18: 170–175. doi:10.1038/s41592-020-01056-5. 33526886 PMC7961889

[24] Wick RR, Judd LM, Gorrie CL Unicycler: Resolving bacterial genome assemblies from short and long sequencing reads. PLoS Comput. Biol., 2017; 13: e1005595. doi:10.1371/journal.pcbi.1005595. 28594827 PMC5481147

[25] Meng G, Li Y, Yang C MitoZ: A toolkit for animal mitochondrial genome assembly, annotation and visualization. Nucleic Acids Res., 2019; 47: 63. doi:10.1093/nar/gkz173. PMC6582343 30864657

[26] Clavijo BJ, Garcia Accinelli G, Wright J W2RAP: A pipeline for high quality, robust assemblies of large complex genomes from short read data. bioRxiv. 2017; 110999. 10.1101/110999.

[27] Mapleson D, Accinelli GG, Kettleborough G KAT: A *k*-mer analysis toolkit to quality control NGS datasets and genome assemblies. Bioinformatics, 2017; 33: 574–576. doi:10.1093/bioinformatics/btw663. 27797770 PMC5408915

[28] Nurk S, Walenz BP, Rhie A HiCanu: Accurate assembly of segmental duplications, satellites, and allelic variants from high-fidelity long reads. Genome Res., 2020; 30: 1291–305. doi:10.1101/gr.263566.120. 32801147 PMC7545148

[29] Wick RR, Schultz MB, Zobel J Bandage: interactive visualization of de novo genome assemblies. Bioinformatics, 2015; 31: 3350–3352. doi:10.1093/bioinformatics/btv383. 26099265 PMC4595904

[30] Manni M, Berkeley MR, Seppey M BUSCO Update: Novel and Streamlined Workflows along with Broader and Deeper Phylogenetic Coverage for Scoring of Eukaryotic, Prokaryotic, and Viral Genomes. Mol. Biol. Evol., 2021; 38: 4647–4654. doi:10.1093/molbev/msab199. 34320186 PMC8476166

[31] Gurevich A, Saveliev V, Vyahhi N QUAST: Quality assessment tool for genome assemblies. Bioinformatics, 2013; 29: 1072–1075. doi:10.1093/bioinformatics/btt086. 23422339 PMC3624806

[32] Guan D, McCarthy SA, Wood J Identifying and removing haplotypic duplication in primary genome assemblies. Bioinformatics, 2020; 36: 2896–2898. doi:10.1093/bioinformatics/btaa025. 31971576 PMC7203741

[33] Jones S, Taylor G, Chan S The Genome of the Beluga Whale (*Delphinapterus leucas*). Genes (Basel), 2017; 8: 378. doi:10.3390/genes8120378. 29232881 PMC5748696

[34] Taylor GA, Kirk H, Coombe L The Genome of the North American Brown Bear or Grizzly: *Ursus arctos* ssp. horribilis. Genes (Basel), 2018; 9: 598. doi:10.3390/genes9120598. 30513700 PMC6315469

[35] Warren RL, Yang C, Vandervalk BP LINKS: Scalable, alignment-free scaffolding of draft genomes with long reads. Gigascience, 2015; 4: 35. doi:10.1186/s13742-015-0076-3. 26244089 PMC4524009

[36] Warren RL, RAILS and Cobbler: Scaffolding and automated finishing of draft genomes using long DNA sequences. J. Open Source Softw., 2016; 1: 116. doi:10.21105/joss.00116.

[37] Li H, Durbin R, Fast and accurate long-read alignment with Burrows-Wheeler transform. Bioinformatics, 2010; 26: 589–595. doi:10.1093/bioinformatics/btp698. 20080505 PMC2828108

[38] Li H, Minimap2: pairwise alignment for nucleotide sequences. Bioinformatics, 2018; 34: 3094–3100. doi:10.1093/bioinformatics/bty191. 29750242 PMC6137996

[39] Kim D, Paggi JM, Park C Graph-based genome alignment and genotyping with HISAT2 and HISAT-genotype. Nat. Biotechnol., 2019; 37: 907–915. doi:10.1038/s41587-019-0201-4. 31375807 PMC7605509

[40] Kim D, Langmead B, Salzberg SL, HISAT: A fast spliced aligner with low memory requirements. Nat. Methods, 2015; 12: 357–360. doi:10.1038/nmeth.3317. 25751142 PMC4655817

[41] Rhie A, Walenz BP, Koren S Merqury: Reference-free quality, completeness, and phasing assessment for genome assemblies. Genome Biol., 2020; 21: 245. doi:10.1186/s13059-020-02134-9. 32928274 PMC7488777

[42] Chen N, Using Repeat Masker to identify repetitive elements in genomic sequences. Curr. Protoc. Bioinformatics, 2004; 5: 4–10. doi:10.1002/0471250953.bi0410s25. 18428725

[43] Smit AFA, Hubley R, RepeatModeler Open-1.0. http://www.repeatmasker.org.

[44] Hubley R, Finn RD, Clements J The Dfam database of repetitive DNA families. Nucleic Acids Res., 2016; 44: D81–D89. doi:10.1093/nar/gkv1272. 26612867 PMC4702899

[45] Bao W, Kojima KK, Kohany O, Repbase Update, a database of repetitive elements in eukaryotic genomes. Mob. DNA, 2015; 6: 1–6. doi:10.1186/s13100-015-0041-9. PMC4455052 26045719

[46] Hoff KJ, Lange S, Lomsadze A BRAKER1: Unsupervised RNA-Seq-based genome annotation with GeneMark-ET and AUGUSTUS. Bioinformatics, 2015; 32: 767–769. doi:10.1093/bioinformatics/btv661. 26559507 PMC6078167

[47] Hoff KJ, Lomsadze A, Borodovsky M Whole-genome annotation with BRAKER. Methods Mol. Biol., 2019; 1962: 65–95. doi:10.1007/978-1-4939-9173-0_5. 31020555 PMC6635606

[48] Brůna T, Hoff KJ, Lomsadze A BRAKER2: automatic eukaryotic genome annotation with GeneMark-EP+ and AUGUSTUS supported by a protein database. NAR Genom. Bioinform., 2021; 3: lqaa108. doi:10.1093/nargab/lqaa108. 33575650 PMC7787252

[49] Danecek P, Bonfield JK, Liddle J Twelve years of SAMtools and BCFtools. Gigascience, 2021; 10(2): giab008. doi:10.1093/gigascience/giab008. 33590861 PMC7931819

[50] O’Leary NA, Wright MW, Brister JR Reference sequence (RefSeq) database at NCBI: Current status, taxonomic expansion, and functional annotation. Nucleic Acids Res., 2016; 44: D733–D745. doi:10.1093/nar/gkv1189. Accessed 15 May 2021. 26553804 PMC4702849

[51] Yates AD, Achuthan P, Akanni W Ensembl 2020. Nucleic Acids Res., 2020; 48: D682–D688. doi:10.1093/nar/gkz966. 31691826 PMC7145704

[52] Dainat J, AGAT : Another Gff Analysis Toolkit to handle annotations in any GTF/GFF format. Zenodo. (Version v0.6.0). 2021; 10.5281/zenodo.4637977.

[53] Jones P, Binns D, Chang HY InterProScan 5: Genome-scale protein function classification. Bioinformatics, 2014; 30: 1236–1240. doi:10.1093/bioinformatics/btu031. 24451626 PMC3998142

[54] Bateman A, Martin MJ, Orchard S UniProt: The universal protein knowledgebase in 2021. Nucleic Acids Res., 2021; 49: D480–D489. doi:10.1093/nar/gkaa1100. Accessed 15 May 2021. 33237286 PMC7778908

[55] Buchfink B, Xie C, Huson DH, Fast and sensitive protein alignment using DIAMOND. Nat. Methods, 2014; 12: 59–60. doi:10.1038/nmeth.3176. 25402007

[56] Buels R, Yao E, Diesh CM JBrowse: A dynamic web platform for genome visualization and analysis. Genome Biol., 2016; 17: 66. doi:10.1186/s13059-016-0924-1. 27072794 PMC4830012

[57] Gremme G, Steinbiss S, Kurtz S, Genome tools: A comprehensive software library for efficient processing of structured genome annotations. IEEE/ACM Trans. Comput. Biol. Bioinform., 2013; 10: 645–656. doi:10.1109/TCBB.2013.68. 24091398

[58] Li H, Tabix: Fast retrieval of sequence features from generic TAB-delimited files. Bioinformatics, 2011; 27: 718–719. doi:10.1093/bioinformatics/btq671. 21208982 PMC3042176

[59] Zhang Z, Schwartz S, Wagner L A greedy algorithm for aligning DNA sequences. J. Comput. Biol., 2000; 7: 203–214. doi:10.1089/10665270050081478. 10890397

[60] Emms DM, Kelly S, OrthoFinder: solving fundamental biases in whole genome comparisons dramatically improves orthogroup inference accuracy. Genome Biol., 2015; 16: 157. doi:10.1186/s13059-015-0721-2. 26243257 PMC4531804

[61] Edgar RC, MUSCLE: multiple sequence alignment with high accuracy and high throughput. Nucleic Acids Res., 2004; 32: 1792–1797. doi:10.1093/nar/gkh340. 15034147 PMC390337

[62] Capella-Gutierrez S, Silla-Martinez JM, Gabaldon T trimAl: A tool for automated alignment trimming in large-scale phylogenetic analyses. Bioinformatics, 2009; 25: 1972–1973. doi:10.1093/bioinformatics/btp348. 19505945 PMC2712344

[63] Kück P, Longo GC, FASconCAT-G: Extensive functions for multiple sequence alignment preparations concerning phylogenetic studies. Front. Zool., 2014; 11: 81. doi:10.1186/s12983-014-0081-x. 25426157 PMC4243772

[64] Nguyen L-T, Schmidt HA, von Haeseler A IQ-TREE: A fast and effective stochastic algorithm for estimating maximum-likelihood phylogenies. Mol. Biol. Evol., 2015; 32: 268–274. doi:10.1093/molbev/msu300. 25371430 PMC4271533

[65] Kalyaanamoorthy S, Minh BQ, Wong TKF ModelFinder: Fast model selection for accurate phylogenetic estimates. Nat. Methods, 2017; 14: 587–589. doi:10.1038/nmeth.4285. 28481363 PMC5453245

[66] Chapman B, Chang J, Biopython: Python tools for computational biology. ACM SIGBIO Newsl., 2000; 20: 15–19. doi:10.1145/360262.360268.

[67] Fonseca E, Machado AM, Vilas-Arrondo N Cartilaginous fishes offer unique insights into the evolution of the nuclear receptor gene repertoire in gnathostomes. Gen. Comp. Endocrinol., 2020; 295: 113527. doi:10.1016/j.ygcen.2020.113527. 32526329

[68] Eide M, Zhang X, Karlsen OA The chemical defensome of five model teleost fish. Sci. Rep., 2021; 11: 1–13. doi:10.1038/s41598-021-89948-0. 34006915 PMC8131381

[69] Li H, Durbin R, Inference of human population history from individual whole-genome sequences. Nature, 2011; 475: 493–496. doi:10.1038/nature10231. 21753753 PMC3154645

[70] Barth JMI, Damerau M, Matschiner M Genomic differentiation and demographic histories of atlantic and indo-pacific yellowfin tuna (*Thunnus albacares*) populations. Genome Biol. Evol., 2017; 9: 1084–1098. doi:10.1093/gbe/evx067. 28419285 PMC5408087

[71] Martins MM, Growth variability in Atlantic mackerel (*Scomber scombrus*) and Spanish mackerel (*Scomber japonicus*) off Portugal. ICES J. Mar. Sci., 2007; 64: 1785–1790. doi:10.1093/icesjms/fsm163.

[72] Satoh TP, Miya M, Mabuchi K Structure and variation of the mitochondrial genome of fishes. BMC Genom., 2016; 17: 719. doi:10.1186/s12864-016-3054-y. PMC5015259 27604148

[73] Hughes LC, Ortí G, Huang Y Comprehensive phylogeny of ray-finned fishes (Actinopterygii) based on transcriptomic and genomic data. Proc. Natl. Acad. Sci. USA, 2018; 115: 6249–6254. doi:10.1073/pnas.1719358115. 29760103 PMC6004478

[74] Rhie A, McCarthy SA, Fedrigo O Towards complete and error-free genome assemblies of all vertebrate species. Nature, 2021; 592: 737–746. doi:10.1038/s41586-021-03451-0. 33911273 PMC8081667

[75] Howe K, Chow W, Collins J Significantly improving the quality of genome assemblies through curation. GigaScience, 2021; 10: 1–9. doi:10.1093/gigascience/giaa153. PMC7794651 33420778

[76] Suda A, Nishiki I, Iwasaki Y Improvement of the Pacific bluefin tuna (*Thunnus orientalis*) reference genome and development of male-specific DNA markers. Sci. Rep., 2019; 9: 1–12. doi:10.1038/s41598-019-50978-4. 31595011 PMC6783451

[77] Santos MM, Ruivo R, Capitão A Identifying the gaps: Resources and perspectives on the use of nuclear receptor based-assays to improve hazard assessment of emerging contaminants. J. Hazard. Mater., 2018; 358: 508–511. doi:10.1016/j.jhazmat.2018.04.076. 29731175

[78] Bertrand S, Thisse B, Tavares R Unexpected novel relational links uncovered by extensive developmental profiling of nuclear receptor expression. PLoS Genet., 2007; 3: 2085–2100. doi:10.1371/journal.pgen.0030188. PMC2065881 17997606

[79] Eide M, Rydbeck H, Tørresen OK Independent losses of a xenobiotic receptor across teleost evolution. Sci. Rep., 2018; 8: 1–13. doi:10.1038/s41598-018-28498-4. 29991818 PMC6039460

[80] López MD, Alcocer MU, Jaimes PD, Phylogeography and historical demography of the Pacific Sierra mackerel (*Scomberomorus sierra*) in the Eastern Pacific. BMC Genet., 2010; 11: 34. doi:10.1186/1471-2156-11-34. 20438637 PMC2876057

[81] Moura AE, Van Rensburg CJ, Pilot M Killer whale nuclear genome and mtDNA reveal widespread population bottleneck during the last glacial maximum. Mol. Biol. Evol., 2014; 31: 1121–1131. doi:10.1093/molbev/msu058. 24497033 PMC3995335

